# Corrigendum

**DOI:** 10.1111/jdi.13487

**Published:** 2021-03-03

The authors would like to draw the reader’s attention to the errors in the following article.

Sakurai K, Kato T, Tanabe H, Naoko Taguchi‐Atarashi N, Sato Y, Eguchi A, Watanabe M, Ohno H, Mori C. Association between gut microbiota composition and glycoalbumin level during pregnancy in Japanese women: Pilot study from Chiba Study of Mother and Child Health. *J Diabetes Investig* 2020; 11: 699–706.

On page 700, the primers used in the 16S rRNA sequencing V1‐2 region were incorrectly stated. The correct primers should have been as follows:

27Fmod: TCGTCGGCAGCGTCAGATGTGTATAAGAGACAGAGRGTTTGATYMTGGCTCAG

338R: GTCTCGTGGGCTCGGAGATGTGTATAAGAGACAGTGCTGCCTCCCGTAGGAGT

The authors apologize for these errors and any confusion they may have caused.

